# Hypomethylation of nerve growth factor (NGF) promotes binding of C/EBPα and contributes to inflammatory hyperalgesia in rats

**DOI:** 10.1186/s12974-020-1711-1

**Published:** 2020-01-24

**Authors:** Hongjie Yuan, Shibin Du, Liping Chen, Xiaoqing Xu, Yufeng Wang, Fuhai Ji

**Affiliations:** 1 The First Affiliated Hospital of Soochow University, Suzhou, 215006 Jiangsu People’s Republic of China; 2 Department of Pain Medicine, Nantong Hospital of Traditional Chinese Medicine, Nantong, 226001 Jiangsu People’s Republic of China; 3 Department of Anesthesiology, Shenzhen University Clinical Medical Academy, Shenzhen University General Hospital, Shenzhen, 518055 Guangdong People’s Republic of China; 4 Department of Radiology, Nantong Hospital of Traditional Chinese Medicine, Nantong, 226001 Jiangsu People’s Republic of China

**Keywords:** NGF, C/EBPα, Inflammatory pain, DNA methylation, Epigenetics

## Abstract

**Background:**

Chronic pain usually accompanied by tissue damage and inflammation. However, the pathogenesis of chronic pain remains unclear.

**Methods:**

We investigated the role of nerve growth factor (NGF) in chronic inflammatory pain induced by complete Freund’s adjuvant (CFA), explored the methylation status of CpG islands in the promoter region of the *NGF* gene, and clarified the function and mechanism of C/EBPα-NGF signaling pathway from epigenetic perspective in the chronic inflammatory pain model.

**Results:**

CFA induced significant hyperalgesia and continuous upregulation of NGF mRNA and protein levels in the L4–6 dorsal root ganglions (DRGs) in rats. Hypomethylation of CpG islands occurred in the *NGF* gene promoter region after CFA treatment. At the same time, the miR-29b expression level was significantly increased, while the DNA methyltransferase 3b (DNMT3b) level reduced significantly. Moreover, CFA treatment promoted binding of C/EBPα to the *NGF* gene promoter region and C/EBPα siRNA treatment obviously decreased expression of *NGF* levels and also alleviate inflammatory hyperalgesia significantly in rats.

**Conclusion:**

Collectively, the results indicated that CFA leads to the upregulation of miR-29b level, which represses the expression of DNMT3b, enhances the demethylation of the *NGF* gene promoter region, and promotes the binding of C/EBPα with the *NGF* gene promoter, thus results in the upregulation of *NGF* gene expression and maintenance of chronic inflammatory pain.

## Background

Chronic pain is usually accompanied by tissue damage and inflammation. Most scholars believe that tissue damage or inflammation leads to increased release of multiple inflammatory factors or pain-related genes, and this may be an important factor for the formation and development of chronic pain [1–3], but the pathogenesis of chronic pain remains unclear.

Based on current research, chronic pain may be involved in epigenetic changes of many coding or non-coding genes. Epigenetic regulation has many forms, such as DNA methylation, histone modification, and non-coding RNA expression [4], in which DNA methylation is the most common. Many studies showed that pain modulation is closely related to epigenetic regulation, especially DNA methylation, which plays an important role in the modulation of inflammation or tissue-damaging pain [5–7]. Further study of the epigenetic changes of related genes in pain modulation may have important clinical significance for the treatment of inflammatory pain, especially chronic pain.

MicroRNAs are a class of endogenous, non-coding RNAs of approximately 22–25 nucleotides in length which have the ability to interfere with protein-encoded mRNA expression. They can bind to the 3′-untranslated region (3′-UTR) region of target mRNAs to regulate gene expression [8]. Recent studies showed that multiple miRNAs are involved in inflammatory pain, such as miR-451 may relieve chronic inflammatory pain through inhibiting microglia activation-mediated inflammation by targeting TLR4 [9], miRNA-219 expression level significantly reduced in CFA-induced chronic inflammation pain in mice model [10], and miR-16 involved in relief of chronic inflammatory pain by targeting RAB23 and inhibiting p38 MAPK activation [11].

Nerve growth factor (NGF) is a member of the neurotrophic factor family, which widely distributed in peripheral Schwann cells, central nervous cells, skeletal muscles, and glands. It can influence the development, differentiation, growth, and survival of peripheral and central nerves and plays an important role in regulating the expression of its functional properties, maintaining sympathetic and sensory nerve fibers and promoting the growth of neuritis [12]. NGF is also a painful inflammatory mediator and plays an important role in inflammation and immune response [13, 14].

CCAAT/enhancer-binding protein α (C/EBPα) belongs to the basic region leucine zipper family of transcription factors, which can promote certain genes expression through interaction with the promoter regions [15]. However, whether C/EBPα regulates the expression of NGF and contributes to inflammatory pain remains unclear.

In the present study, we explored the regulatory mechanism of NGF in regulating CFA-induced inflammatory pain from an epigenetic perspective. We found that there are two CpG islands in the promoter region of *NGF* gene by online analysis, and the ratio of methylated and unmethylated CpGs in the *NGF* gene promoter region was significantly altered in the DRGs of rats with inflammatory pain. The results indicated DNA methylation may participate in regulating CFA-induced inflammatory pain. Further, CFA treatment induced demethylation of the *NGF* gene promoter region and promoted binding of C/EBPα with the *NGF* gene promoter. Finally, C/EBPα siRNA treatment downregulated the expression level of NGF as well as alleviated inflammatory hyperalgesia in rats.

## Materials and methods

### Animal experimental model establishment

Male SD rats from the Experimental Animal Center of Soochow University weighing about 180 g were all clean grade. Standardized breeding was carried out at the Experimental Animal Center of Soochow University and light/dark of 12-h cycle was carried out. The ambient temperature was maintained at 25 °C. Establishment of inflammatory pain models as described previously [16]. In brief, by subcutaneous injection of 0.1 mL of CFA on the unilateral plantar skin, we established the model of chronic inflammatory pain. All experiments were approved by the Animal Research Ethics Committee from Soochow University.

### Measurement of mechanonociceptive threshold

The measurement of mechanonociceptive threshold by von Frey filaments (vFF 0.4 g–15.0 g) in rats is commonly known as the paw withdrawal threshold (PWT). Before the behavioral experiment, the rats were placed in a 24 cm × 12 cm × 24 cm plexiglass box on a screen mesh for 3 days, 1 h a day. The up-and-down method was used to estimate the threshold of 50% contraction: firstly, the rats were placed in the box for half an hour, and then a series of calibrated von Frey filaments were used to vertically stimulate the plantar surface of the hind paw, with the filament bent for 1 to 2 s. If the rats had lameness or foot-lifting behavior, it was considered a positive reaction, and the opposite was considered a negative reaction. The measurement starts from 6 g first; if the stimulation does not cause a positive reaction, then it gives a large level of intensity to stimulate, and if a positive reaction occurs, then a filament of the next lower force was applied and then repeated until the first positive occurs. The junction value of the positive and negative reactions should be continued for four times, with an interval of 5 min. Finally, it can be obtained that more than three positive reactions in five consecutive stimulations are the lowest stimulation intensity, which can be considered as the threshold of the PWT of the rat. Set 15 g as the maximum intensity to avoid damage to the skin in the test rats. Each trial was repeated three times at 10-min intervals, and the mean value was used as the force to produce a withdrawal response.

### Measurement of thermonociceptive threshold

Thermonociceptive threshold was measured by thermal radiation method to evaluate thermal hyperalgesia in model rats, commonly known as paw withdrawal latency (PWL). The plexiglass boxes were firstly placed on a glass plate, and then the rats were placed in these boxes for half an hour. A radiant heat source from a thermal stimulator was concentrated on the plantar surface of the hind paw according to the Hargreaves method [17]. The time from the start of the irradiation to the occurrence of the leg-retraction was defined as PWL. Set the maximum exposure time to 20 s to avoid tissue damages. Three PWL measurements were performed on each rat at intervals of 10 min and were averaged as the result of each test session. All these behavioral experiments were conducted by using double-blind methods.

### Cell culture and DRG isolation

PC12 cell line was purchased from the American Type Culture Collection (ATCC, Manassas, VA, USA). The cell line was cultured with Ham’s F12K that included 10% FBS (Invitrogen), 100 U/mL penicillin, and 100 μg/mL streptomycin. Lipofectamine 3000 Transfection Reagent was purchased from Invitrogen Corporation, and the operation was in accordance with the operating instructions strictly. Isolation of DRG neurons from SD rats is as described previously [6]. Briefly, at each time point after injection of CFA or saline, the rats were killed followed by decapitation. Lumber DRGs (L4–L6) were dissected out and transferred into an ice-cold fresh dissecting solution for mRNA and protein extraction.

### Real-time quantitative PCR

Expressions of mRNA and miRNA in DRG samples from control and CFA-treated rats were measured by real-time quantitative PCR analyses. Rats were firstly euthanized by an overdose of pentobarbital. Lumbar DRGs (L4–6) were quickly dissected out and frozen in liquid nitrogen. Total RNA was extracted from DRGs using Trizol reagent (Invitrogen) according to the manufacturer’s protocol. The primer sequences (miR-29b, C/EBPα, NGF, DNMT3a, DNMT3b, and β-actin) were listed in Table 1. The qPCR amplifications were simply performed as follows: 95 °C for 15 s and 60 °C for 45 s with 40 cycles. A Ct (cycle threshold) value corresponding to the amplification curve was obtained after completion of the PCR reaction. The relative expression level for each target gene was normalized by the Ct value of β-actin or U6 were quantitatively analyzed by using the 2^−ΔΔCt^ method.

**Table 1:** Primers used in qPCR

Gene	Primer sequence
miR-29b-F	5′-ACACTCCAGCTGGGTGATTGTCCAAACGC-3′
miR-29b-R	5′-TGGTGTCGTGGAGTCG-3′
U6-F:	5′-CTCGCTTCGGCAGCACA-3′
U6-R:	5′-AACGCTTCACGAATTTGCGT-3′
C/EBPα-F	5′-TTCCAAGGGTGTATGTAGTTGTGG-3′
C/EBPα-R	5′-GCTTCCAGTGGCAGGGTTG-3′
NGF-F:	5′-AGGCTTTGCCAAGGACG-3′
NGF-R:	5′-CCAGTGGGCTTCAGGGA-3′
β-actin-F:	5′-GATGGAAAGTGACCCGCA-3′
β-actin-R:	5′-GAGGAAGACGCAGAGGTTTG-3′
DNMT3a-F:	5′-GAGGGAACTGAGACCCCAC-3′
DNMT3a-R:	5′-CTGGAAGGTGAGTCTTGGCA-3′
DNMT3b-F:	5′-CATAAGTCGAAGGTGCGTCGT-3′
DNMT3b-R:	5′-ACTTTTGTTCTCGCGTCTCCT-3′

### Western blot

The DRGs were homogenated with tissue lysate plus protease inhibitors according to the manufacturer’s instruction (Promega, Madison, WI, USA), and then centrifuged and collected the supernatant and determined concentration of the protein (Bio-Rad, Hercules, CA, USA). By SDS-PAGE gel, the protein samples (30 μg) were separated and transferred to a polyvinylidene fluoride (PVDF) membrane. The membrane was blocked with 5% milk and then incubated with rabbit anti-NGF antibody at 4 °C overnight (1:1000, ab6199, Abcam), rabbit anti-C/EBPα antibody (1:1000, ab40764, Abcam), rabbit anti-DNMT3a antibody (1:800, ab4897, Abcam), rabbit anti- DNMT3b antibody (1:1000, ab2851, Abcam), or mouse anti-β-actin antibody (1:5000, ab8227, Abcam). After that, the membrane was also incubated with secondary antibodies (1:10000, goat anti-mouse IgG (H + L) or donkey anti-rabbit IgG (H + L) labeled with IRDye 800CW). The blot intensity was analyzed using Odyssey (Li-COR, USA) for the gray scale value statistics. The protein expression was normalized to β-actin.

### Methylation-specific and bisulfite sequencing PCR

Methylation-specific PCR (MSP) and bisulfite sequencing PCR (BSP) determined the methylation status of CpG islands in the *NGF* gene promoter region. Extracted DNA from L4–6 DRGs modified with bisulfite (Zymo Research, Orange, CA) leads to the change of unmethylated cytosine to thymine. A total of 20 ng of bisulfite-modified DNA was subjected to PCR amplification and directly sequenced using an automated sequencing system ABI3700 (Applied Biosystems, CA, USA). The methylated (M) band designates the CpG sites were in the methylation status by MSP analysis and the unmethylated (U) band represents the status of unmethylation. Bisulfite sequencing PCR was also the method to verify the methylation status of the CpG islands. In brief, bisulfite-treated DNA was amplified by PCR. Then the PCR products were purified using a TIANgel Midi Purification Kit (Tiangen Biotech Co. Ltd). After that, the PCR products were cloned into a pGEM-T easy vector (Promega, Madison, WI, USA). Finally, 12 colonies were selected randomly for the plasmid extraction using a Promega Spin Mini kit (Promega) and sequenced by a Genetic Analyzer ABI 3130 (Applied Biosystems, Foster City, CA, USA). The detailed specific primer sequences designed for the *NGF* gene were listed in Table 2.

**Table 2:** Primers used in Methylation-specific PCR and bisulfite sequencing

Gene	Primer sequence
MSP	
NGF-MSP-M-F1:	5′-GAGTCGAGTTTTATAAGGGTGTAGC-3′
NGF-MSP-M-R1:	5′-ACTTCTTTCAAAAATACGTTTCGAC-3′
NGF-MSP-U-F1:	5′-TTGAGTTGAGTTTTATAAGGGTGTAGT-3′
NGF-MSP-U-R1:	5′-ACTTCTTTCAAAAATACATTTCAAC-3′
BSP	
NGF-BSP-F:	5′-GAGTTTGGAGGAGGGGTTAAATATA 3′
NGF-BSP-R:	5′-CCAAACAAAAAACCAAACACAC-3′

### Dual luciferase reporter assay

In brief, the wild-type DNMT3b-3′UTR and mutant DNMT3b-3′UTR were cloned into the XbaI site of the pGL3-basic plasimds (Promega), respectively. Cells (1 × 10^5^) were seeded into the 24-well plates to culture for 24 h. Then the cells were transfected with either the pGL3-wt-DNMT3b-3′-UTR or pGL3-mut-DNMT3b-3′-UTR vectors, together with the pRL-TK renilla plasmids (Promega) and the miR-29b mimic or miR-con by using lipofectamine 3000 transfection reagent (Invitrogen). After 48 h of transfection, the activity of the reporter was measured by using a Dual Luciferase Reporter Assay Kit (Promega) and the relative luciferase activities were determined.

### Chromatin immunoprecipitation assay

Chromatin immunoprecipitation (ChIP) was determined using ChIP Enzymatic Chromatin IP Kit (Magnetic beads, Cell Signaling, Danvers, MA, USA). In brief, the tissues were first cut into small pieces, and then the protein-DNA complexes were crosslinked with 1% formaldehyde followed by nuclear fractionation and DNA shearing through sonication. Then the purified chromatin was immunoprecipitated using an anti-C/EBPα antibody (sc-365318, Santa Cruz Biotechnology) or mouse immunoglobulin G (IgG, negative control). The antibody–protein–DNA complex was eluted from the beads, and the crosslinking was reversed by incubation after washing. The purified DNA was subjected to PCR using primers specific to the rat *NGF* gene promoter after removing the protein and RNA. The PCR conditions were as follows: 95 °C for 5 min, 35 cycles (95 °C for 30 s, 54–60 °C for 30 s, and 72 °C for 30 s), and 72 °C for 10 min. ChIP primers for detailed sequences were listed in Table 3.

**Table 3:** Primers used in ChIP PCR

Gene	Primer sequence
NGF/C/EBPα-321-F	5′-TGGTCGTGAAATTCCCTGTC-3′
NGF/C/EBPα-321-R	5′-ATCCCTCACTCCAGGCTCTC-3′
NGF/C/EBPα-492-F	5′-GCTAGGGTGGCGGAGCAGT-3′
NGF/C/EBPα-492-R	5′-CGGCGACTGGTCCTCTTACC-3′

### Drugs administration

To investigate the role of C/EBPα, C/EBPα siRNA was used in this study. Male Sprague-Dawley rats of about 180 g were divided into four groups (four per group) and injected subcutaneously with 0.1 mL of CFA on the right foot. 5′-cholesteryl modified and 2′-O-methyl-modified C/EBPα small interfering RNA (siRNA; 5′-GGAGTTGACCAGTGACAAT-3′) and an additional scrambled siRNA were purchased from RiboBio. Intrathecal injection was made with a 30-G needle between the L5 and L6 intervertebral spaces to deliver the reagents to the cerebrospinal fluid after CFA injection. PWT and PWL were recorded 1 day, 2 days, 3 days, and 4 days after C/EBPα siRNA treatment, respectively.

### Data analysis

Data were represented as mean ± SD. Behavioral data were analyzed by two-way repeated measures ANOVA. The levels of NGF, DNMT3a, DNMT3b, and C/EBPα were normalized to loading control β-actin. Differences between groups were compared using one-way ANOVA followed by the Bonferroni test. Student’s *t* test was used if there are only two groups for comparison. *P* < 0.05 was considered statistically significant.

## Results

### CFA-induced inflammatory pain model of rats

**Fig. 1:**
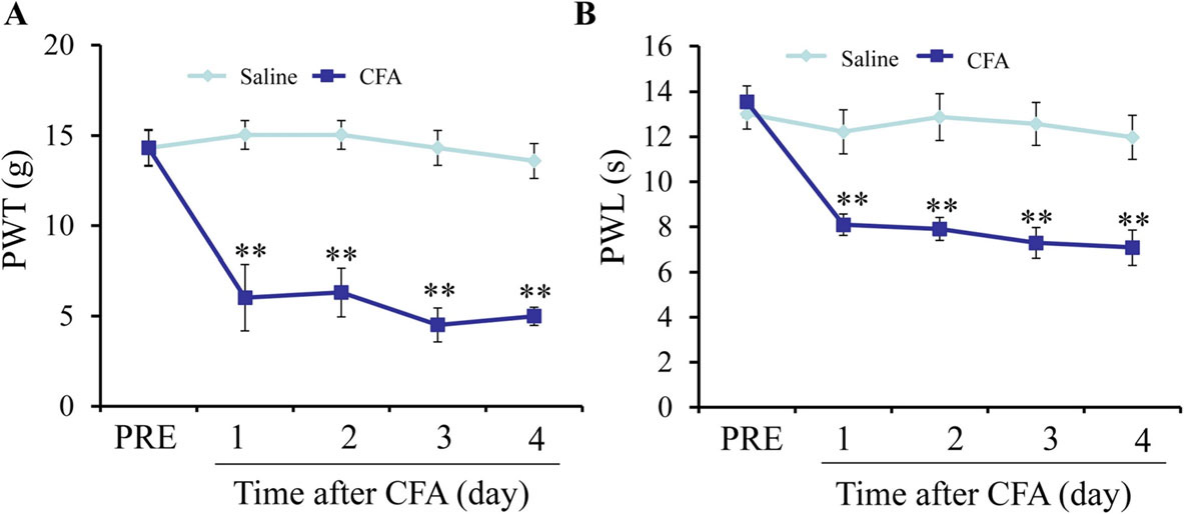
Dynamic changes of paw withdrawal threshold (PWT) and paw withdrawal latency (PWL) after CFA treatment. **a** 1 day after injection, the PWT of the CFA injection group (CFA) was significantly decreased (**P < 0.01, compared with the corresponding saline control group (Saline), *n* = 7). **b** The PWL was also significantly decreased in the CFA group compared to the corresponding saline-treated control group starting at 1 day after injection (**P < 0.01, *n* = 7)

To observe the CFA-induced inflammatory pain behavior in rats, we found injection of CFA produced a significant reduction in paw withdrawal threshold (PWT) starting at 1 day after injection (**P < 0.01, Fig. 1a). The paw withdrawal latency (PWL) also shortened obviously in the CFA group than in the saline group 1 day later (**P < 0.01, Fig. 1b).

### Upregulation of NGF expression in DRGs after CFA treatment

**Fig. 2:**
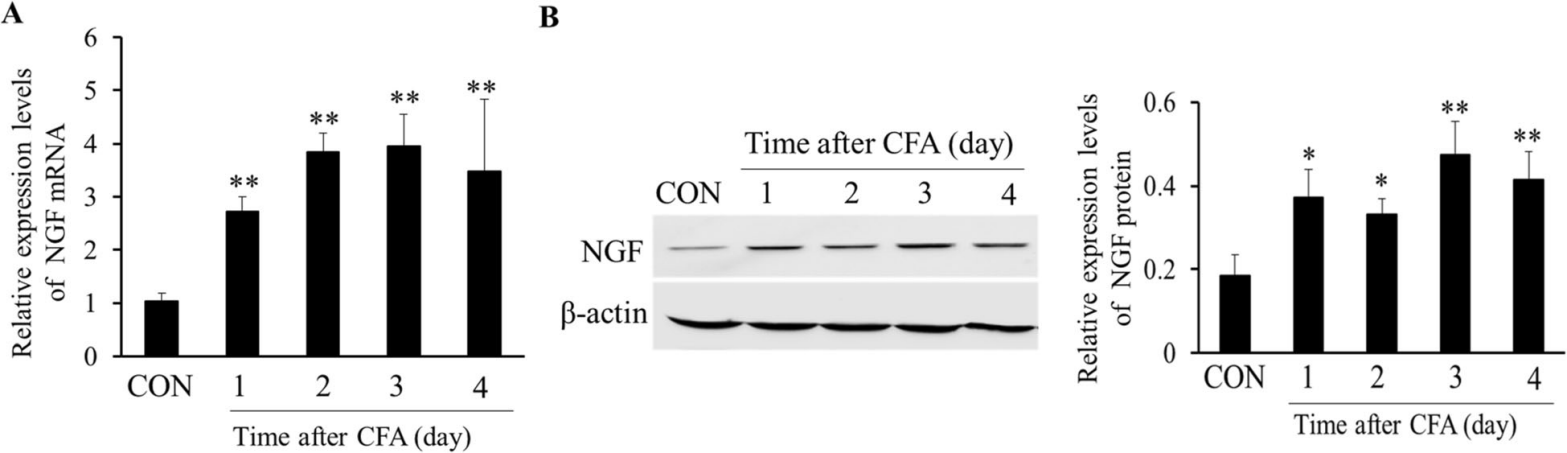
Upregulation of NGF expression in DRGs after CFA treatment. **a**, **b** NGF mRNA and protein expression were all increased significantly compared with the saline-treated group (CON) after CFA injection (*P < 0.05, **P < 0.01 compared with CON group, *n* = 4)

We detected the relative expression levels of the *NGF* gene and protein in the L4–6 DRGs before the saline-treated group (CON) and at 1, 2, 3, and 4 days after CFA treatment. The results showed that CFA induced continuous upregulation of NGF mRNA, which started at the time point of 1 day, peaked at 3 days, and still increased at the time point of 4 days (**P < 0.01, Fig. 2a). Similar to the relative expression of NGF mRNA in each time point, the protein level of NGF was also elevated significantly 1 day after CFA treatment and peaked at 3 days (*P < 0.05, **P < 0.01, Fig. 2b).

### Hypomethylation of CpG islands in the *NGF* gene promoter after CFA treatment

**Fig. 3:**
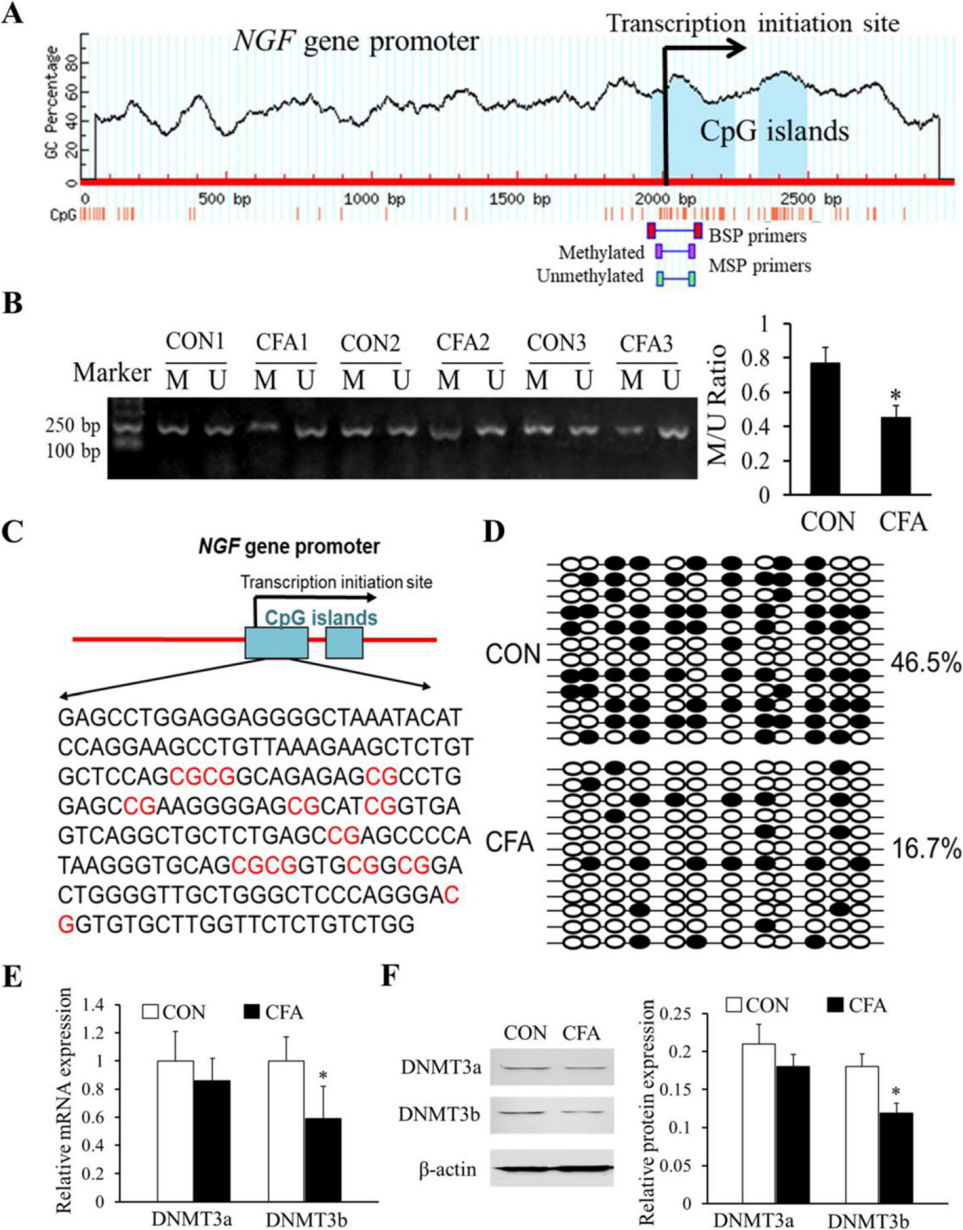
Hypomethylation of CpG islands of the *NGF* gene promoter after CFA treatment. **a** Online software prediction of CpG islands around the *NGF* gene promoter region. The light blue areas on the map indicate the potential CpG islands. **b** The representative MSP assay showed DNA methylation and unmethylation ratio of the CpG islands in the *NGF* gene promoter region of L4–6 DRGs was significantly decreased after 3 days of CFA treatment (*P < 0.05, compared with the control group, *n* = 4). **c** Schematic of CpG island showing locations of the 12 CpG sites in the *NGF* gene promoter area **d** BSP sequencing, CFA injection resulted in a significant percentage of methylated CpG sites decrease to 16.7% in CpG islands of the *NGF* gene promoter region. **e**, **f** The expression of DNMT3b mRNA and protein in DRGs were significantly decreased after CFA treatment (3 days after CFA injection) (*P < 0.05, compared with the control group, *n* = 4), while DNMT3a level did not change significantly

To explore whether DNA hypomethylation leads to the upregulation of NGF, we detected the methylation status in the *NGF* gene promoter region by using the method of methylation-specific PCR (MSP) and bisulfite sequencing PCR (BSP) assays. We firstly predicted the underlying CpG islands in the promoter region of the *NGF* gene by the online software (http://www.urogene.org/cgi-bin/methprimer/methprimer.cgi) and found that there were two potential CpG islands around the promoter region of the NGF genomic structure (Fig. 3a). The result of MSP assay showed significant hypomethylation in the promoter region of the *NGF* gene in the CFA-treated group after 3 days of treatment (*P < 0.05, Fig. 3b). Moreover, BSP results also verified that the methylation degree of the CpG sites in the promoter region of the *NGF* gene was reduced after CFA-induced inflammatory pain (Fig. 3c, d).

Further, we detected the expressions of DNA methyltransferases (DNMT3a and DNMT3b) by qPCR and western blot. The results indicated that DNMT3b mRNA as well as the protein, not DNMT3a, decreased significantly after 3 days of CFA treatment (*P < 0.05, Fig. 3e, f).

### DNMT3b is one of the targets of miR-29b

**Fig. 4:**
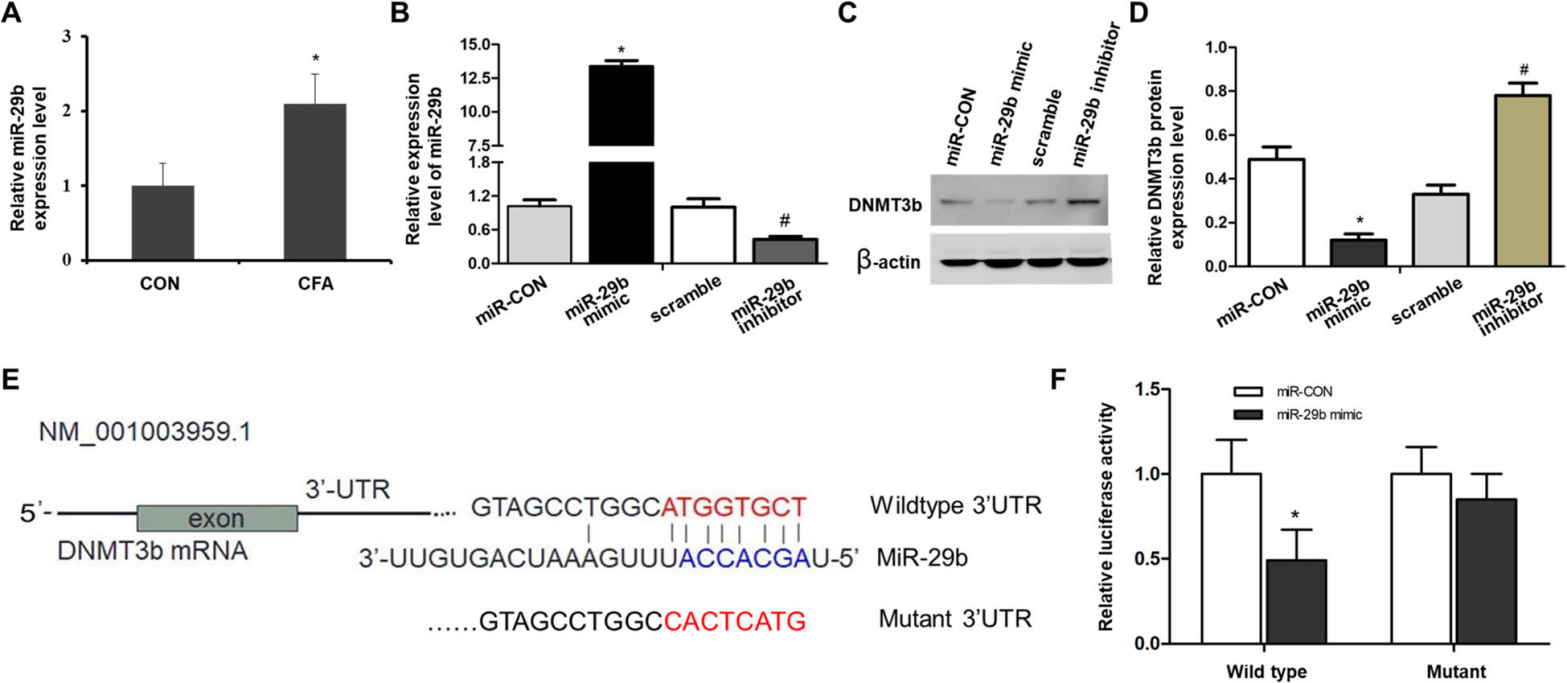
DNMT3b is a direct target of miR-29b in DRGs. **a** The expression of miR-29b was significantly increased in DRGs after CFA treatment (*P < 0.05, compared with the control group, n = 4). **b** The relative expression level of miR-29b when transfected with its inhibitor and mimic compared with scramble or miR-CON group, respectively (*P < 0.05, #*P* < 0.05). **c**,**d** Western blot analysis showed that overexpression of miR-29b inhibited the protein level of DNMT3b, and inhibition of miR-29b expression promoted the expression of DNMT3b protein (*P < 0.05, #*P* < 0.05). **e** The reverse binding sequence of mature miR-29b inserted into C-terminal of the luciferase gene to generate pGL3-DNMT3b -3’UTR. **f** miR-29b targeted the wild type but not the mutant 3’UTR of DNMT3b (*P < 0.05)

To explore the mechanism of DNMT3b expression decline, we predicted that there was a potential binding site of miR-29 at the DNMT3b mRNA 3′UTR through online software TargetScan 6.2 and miRanda 3.2 (Fig. 4e). Then we detected the relative expression level of miR-29b in CFA-induced pain model and found it was significantly increased in DRGs after CFA treatment (*P* < 0.05, Fig. 4a). Further, by using luciferase activity assay method, we found that the overexpressed miR-29b inhibited the luciferase activity of pGL3-DNMT3b-3′-UTR reporter, while not reducing the luciferase activity of pGL3-mut-DNMT3b-3′-UTR (*P* < 0.05, Fig. 4f). At the same time, we verified that the PC12 cell line, transfected with miR-29b mimic or inhibitor, significantly decrease or increase its level, respectively (*P* < 0.05, Fig. 4b). Moreover, western blot indicated that DNMT3b protein levels were decreased significantly when miR-29b mimic was transfected in the PC12 cell (*P* < 0.01, Fig. 4c, d).

### Promoted binding of C/EBPα to the *NGF* gene promoter region after CFA treatment

**Fig. 5:**
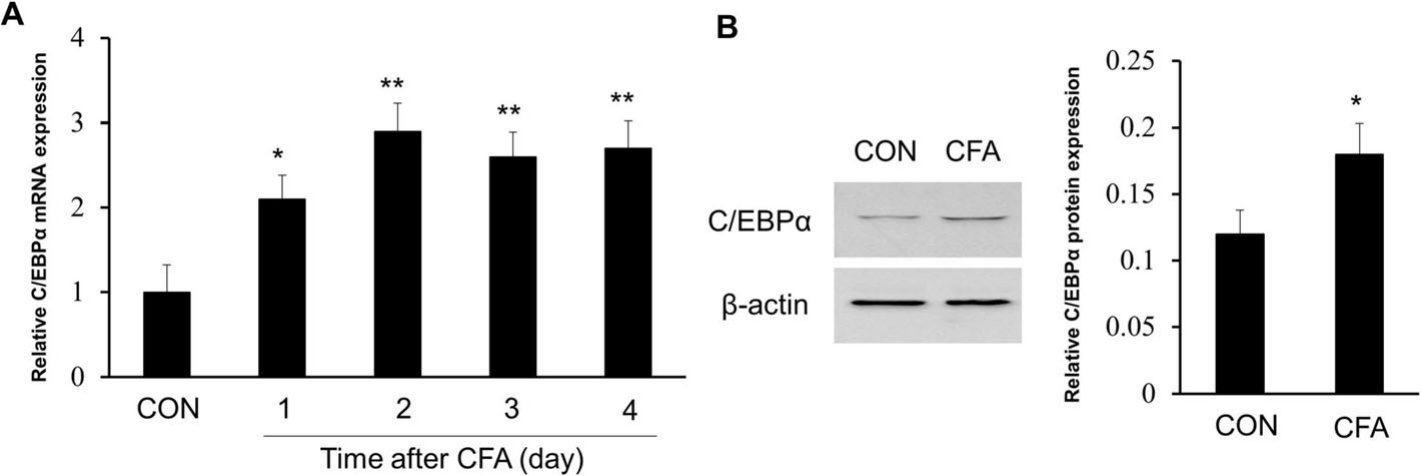
Relative C/EBPα expression levels are upregulated significantly in DRGs after CFA treatment. **a**, **b** Relative C/EBPα mRNA and protein expressions were all increased significantly compared with the saline-treated group (CON) after 1 day of CFA injection (*P < 0.05, **P < 0.01 compared with CON group, *n* = 4)

**Fig. 6:**
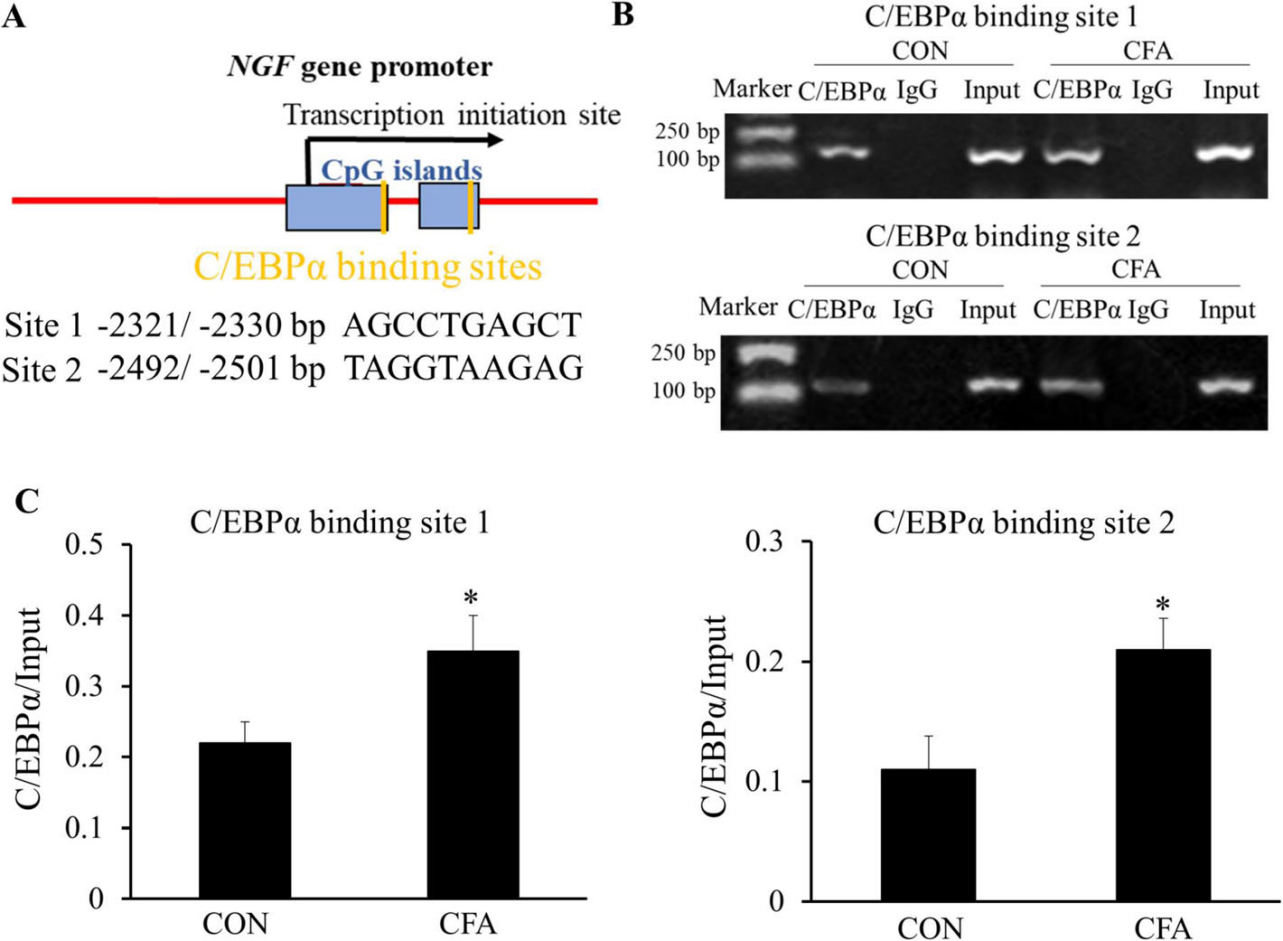
Promoted binding of C/EBPα to the *NGF* gene promoter region in DRGs after CFA treatment. **a** Two predicted C/EBPα binding sites in the CpG islands of the *NGF* gene promoter. **b**, **c** Chromatin immunoprecipitation assays indicate a significant increase in binding activity of C/EBPα with the two sites of *NGF* gene promoter in CFA-induced inflammatory rats when compared with control rats (*P < 0.05, compared with the control group, *n* = 4 for each group)

To further explore the upregulation mechanism of NGF expression, we found two potential C/EBPα binding sites in the promoter region of the *NGF* gene through online prediction (http://gene-regulation.com) (Fig. 6a). C/EBPα belongs to a family of transcription factors and participated in the regulation of multiple functions widely such as inflammation and innate immunity [18, 19]. Therefore, we explored if C/EBPα was participated in regulating the expression of NGF. Firstly, we detected the C/EBPα gene as well as protein expression levels in the L4–6 DRGs at 1, 2, 3, and 4 days, respectively, after CFA treatment. The results showed that CFA induced continuous upregulation of C/EBPα mRNA (*P < 0.05, **P < 0.01, Fig. 5a) and the upregulated C/EBPα protein levels at 4 days (*P < 0.05, Fig. 5b). Further, the chromatin immunoprecipitation (ChIP) data showed that after 3 days of CFA treatment, the two binding sites all occurred enhanced binding of C/EBPα to the promoter region of the *NGF* gene (*P < 0.05, Fig. 6b, c).

### C/EBPα siRNA reversed expression level of NGF and alleviated inflammatory hyperalgesia

**Fig. 7:**
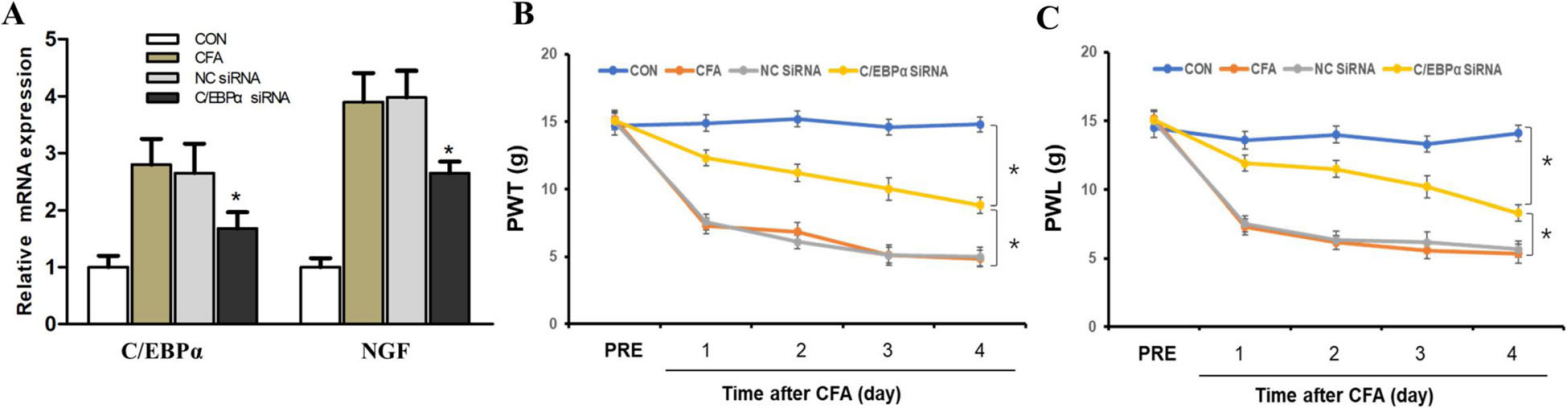
C/EBPα siRNA downregulates the expression level of NGF. **a** C/EBPα siRNA treatment obviously reverses the expression of NGF levels when 3 days after CFA treatment (*P < 0.05, compared with NC siRNA group, *n* = 4 for each group). **b**, **c** C/EBPα siRNA significantly alleviate inflammatory hyperalgesia in rats that induced by CFA (*P < 0.05, **P < 0.01, compared with NC group, *n* = 4 for each group)

To further determine whether there is a mutual regulation relationship between C/EBPα and NGF, we examined whether injection of C/EBPα siRNA could inhibit the expression level of NGF in rats after CFA treatment. The results showed C/EBPα siRNA treatment obviously decreased the expression of the NGF level 3 days after CFA treatment though not bringing the C/EBPα and NGF expression down to the control (*P < 0.05, Fig. 7a), and the treatment is effective obviously for it significantly alleviated inflammatory hyperalgesia in rats that are induced by CFA (*P < 0.05, Fig. 7b, c).

## Discussion

In this study, we attempt to clarify epigenetic regulations of the *NGF* gene expression and a novel role for C/EBPα signaling in rats with CFA-induced chronic inflammatory pain hypersensitivity. We provided direct evidence to support that C/EBPα signaling may contribute to the development of chronic inflammatory pain by activation of the *NGF* gene.

**Fig. 8:**
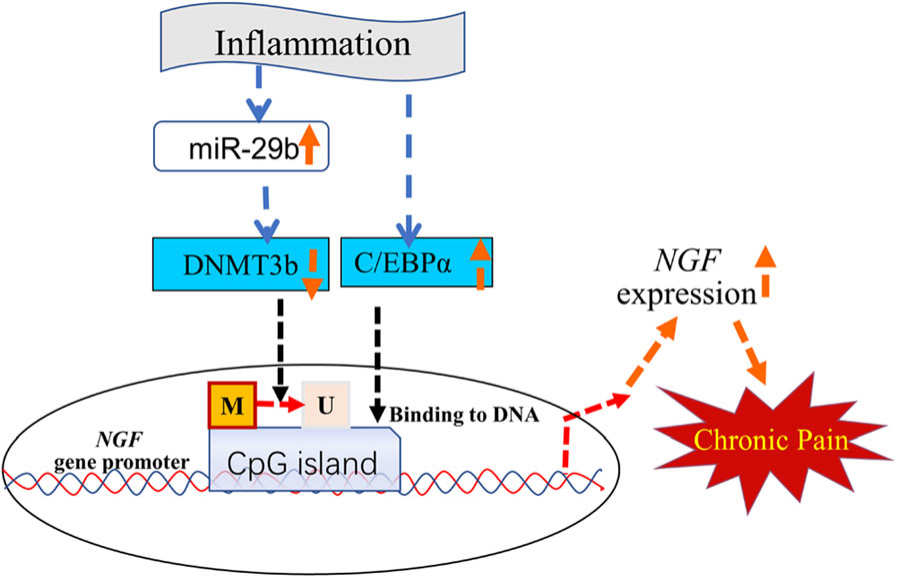
Schematic shows the regulation of the *NGF* gene expression and the involvement of C/EBPα in chronic inflammatory pain**.** Chronic inflammation leads to upregulation of miR-29b expression level which represses the activity of DNMT3b, enhances the demethylation of the *NGF* gene promoter region, and promotes the binding of C/EBPα with *NGF* gene promoter, resulting in upregulation of *NGF* gene expression, and contribute to the maintenance of chronic inflammatory pain

The CFA-induced inflammatory pain model is one of the commonly used animal models of chronic inflammatory pain as the mechanical allergy induced by CFA can last for quite a long time, and our experimental results showed in Fig. 1 were consistent with the previous reports [11, 20–22]. Some studies reported that CFA injection can significantly increase histone deacetylase HDACs expression and induce inflammatory pain, while the HDAC blockers can significantly alleviate CFA-induced inflammatory pain [23, 24]. Accumulating studies have shown that NGF is involved in the pathophysiological process of pain [25, 26]. However, the mechanism by which NGF is upregulated in a CFA-induced inflammatory pain has not been well studied. Our study clarifies for the first time that DNA demethylation of the NGF promoter region in DRGs is associated with the upregulation of NGF after CFA treatment. The results further affirmed that chronic inflammatory pain leads to the upregulation of miR-29b level, which represses the activity of DNMT3b, enhances the demethylation of the *NGF* gene promoter region, promotes the binding of C/EBPα with NGF, and thus results in the upregulation of the *NGF* gene expression (Fig. 8). Moreover, C/EBPα siRNA treatment obviously downregulates expression of NGF levels and significantly alleviates inflammatory hyperalgesia in rats that are induced by CFA. Thus, our results demonstrate that NGF is involved in mediating chronic inflammatory pain from an epigenetic perspective.

It is because by which mechanism C/EBPα binding to the *NGF* gene was enhanced remains unknown, we therefore propose a mechanism that a significant DNA demethylation of NGF promoter is a basis for the enhanced binding activity. Epigenetic regulation in gene expression involves various epigenetic modifications, including DNA methylation, gene silencing, and RNA editing [27]. DNA methylation as a heritable epigenetic modification is the process of selectively adding a methyl group to cytosine to form 5-methylcytosine under the action of DNA methyltransferases (DNMTs) [28]. Studies have reported that DNA methylation is associated with inflammatory pain [29, 30]. Our results showed that CFA induced continuous upregulation of NGF mRNA in DRGs, with hypomethylation of CpG islands of the *NGF* gene promoter region, indicated that DNA methylation was involved in the regulation of NGF expression in DRGs after CFA treatment.

MiR-29b is one of the members of the miR-29 family. The family consists of miR-29a, miR-29b, and miR-29c, which is encoded and transcribed by two genes located on chromosome 7q32.3 or chromosome 1q32.2 in tandem, respectively. Recent studies showed that miR-29 participated in the regulation of many diseases, such as glioblastoma multiforme [31], pulmonary fibrosis [32], and gastric cancer [33]. In this study, we found that miR-29b is also involved in CFA-mediated inflammatory responses and can target DNA methyltransferases (DNMT3b), which provides more detailed evidence for the study of the regulation mechanism of genes that could illuminate inflammatory pain from an epigenetic perspective. As we all know, DNMTs mediate DNA methylation mainly, including DNMT3a and DNMT3b [34, 35]. In this paper, we explored DNMT3a and DNMT3b expression in the CFA-induced chronic inflammation group and found that the DNMT3b downregulation could be the main reason for the hypomethylation of the NGF promoter region under inflammatory pain.

C/EBP is a family of transcription factors that all contain a highly conserved, basic-leucine zipper domain at the C-terminus that is involved in dimerization and DNA binding. This family of transcription factors regulate viral and cellular CCAAT/enhancer element-mediated transcription which consist of several related proteins, C/EBPα, β, γ, δ that form homodimers or heterodimers with each other [19]. In this study, we discovered two potential sites for C/EBPα binding and confirmed the critical role of C/EBPα in regulating inflammatory pain by regulating NGF expression. Interestingly, prior work has suggested that C/EBPα is a neuronal transcriptional regulator that can be activated by NGF receptor signaling [36], and we are interested if the activated C/EBPα induced by NGF upregulation can combine with C/EBPα and further enhance binding of C/EBPα to the NGF promoter region, and thus form a positive feedback path. We will conduct further research at this point.

## Conclusions

Chronic inflammation leads to upregulation of miR-29b and, by targeting DNMT3b, enhances the demethylation of the *NGF* gene promoter region, and thus promotes the binding of C/EBPα with the *NGF* gene promoter, resulting in the upregulation of the *NGF* gene expression. C/EBPα siRNA treatment significantly downregulated the expression of NGF levels and can also significantly alleviate inflammatory hyperalgesia in rats that are induced by CFA. The results may provide new strategies of treatment for chronic inflammatory pain from epigenetic perspective.

## Data Availability

The datasets used and/or analyzed during the current study are available from the corresponding author on reasonable request.

## Abbreviations

3′-UTR: 3′-Untranslated region
BSP: Bisulfite sequencing PCR
CFA: Complete Freund’s adjuvant
ChIP: Chromatin immunoprecipitation
DNMT3a: DNA methyltransferase 3a
DNMT3b: DNA methyltransferase 3b
DRGs: Dorsal root ganglions
MSP: Methylation-specific PCR
NGF: Nerve growth factor
PVDF: Polyvinylidene fluoride
PWL: Paw withdrawal latency
PWT: Paw withdrawal threshold
vFF: Von Frey filaments
